# Advances in the immunological microenvironment and immunotherapy of bladder cancer

**DOI:** 10.3389/fimmu.2025.1609871

**Published:** 2025-08-19

**Authors:** Le Meng, Xiangyu Zhu, Xuran Ji, Bowen Wang, Haoxun Zhang, Guoling Zhang, Yue Xue, Chunyang Wang

**Affiliations:** Department of Urology, the First Affiliated Hospital of Harbin Medical University, HeiLongJiang Harbin, China

**Keywords:** bladder cancer, immunotherapy, immune checkpoint inhibitors, tumor microenvironment, PD-1/PD-L1, CAR-T cells

## Abstract

Bladder cancer remains a significant global health challenge, particularly affecting male populations. While radical cystectomy and chemotherapy have been mainstays of treatment, their substantial morbidity and impact on quality of life have driven the development of bladder-preserving immunotherapeutic strategies. Clinical trial data support the use of ICIs as first-line therapy for cisplatin-ineligible patients, second-line treatment for platinum-refractory disease, and maintenance therapy. This review comprehensively summarizes the advances in bladder cancer immunotherapy, focusing on the tumor immune microenvironment and emerging treatment modalities, as well as the roles of immune checkpoint inhibitors (ICIs) targeting PD-1/PD-L1 and CTLA-4 pathways, which have demonstrated remarkable efficacy in both muscle-invasive (MIBC) and non-muscle invasive bladder cancer (NMIBC). This review also provides novel approaches including combination immunotherapies, tumor vaccines, adoptive cellular therapies, and oncolytic viruses. Overall, these immunotherapeutic advances are transforming bladder cancer management, offering improved outcomes while reducing treatment morbidity.

## 1 Introduction

Bladder cancer remains one of the most common malignancies among male populations (1, 2). Conventional treatment modalities, such as radical cystectomy and neoadjuvant chemotherapy, are associated with considerable morbidity and a profound impact on patients’ quality of life, prompting increasing interest in bladder-preserving therapeutic approaches (3, 4). While radical cystectomy demonstrates favorable oncological control, high recurrence rates and suboptimal five-year survival rates persist—even in cases with negative surgical margins and lymph node involvement—highlighting the urgent demand for novel anti-tumor strategies (5).

Recent advancements in immunotherapy have revolutionized the therapeutic paradigm for bladder cancer. Immune checkpoint inhibitors, particularly those targeting CTLA-4 and PD-1/PD-L1 pathways, play a crucial role in counteracting tumor immune evasion mechanisms (6, 7). These developments not only enhance treatment efficacy but also provide valuable insights into the mechanisms underlying tumor immune escape. Key approaches include immune checkpoint inhibitors, tumor vaccines, adoptive cellular immunotherapy, oncolytic immunotherapy, and biological response modifiers. Among these, CAR-T cell therapy and immune checkpoint inhibitors have demonstrated particularly promising clinical outcomes (8, 9). This review synthesizes current research on the immunological microenvironment and immunotherapy in bladder cancer, with a focus on strategies designed to reactivate the immune system against tumor cells. Besides, this review further provides evidence-based insights and potential directions for future bladder cancer treatment.

## 2 Immune microenvironment of bladder cancer

The tumor microenvironment (TME) consists of malignant cells, immunomodulatory components, and stromal elements, with the immune compartment exerting a profound influence on disease progression (10–12). In urothelial carcinoma, major immune effectors include CD4^+^ T helper cells, cytotoxic CD8^+^ T lymphocytes (CTLs), dendritic cells (DCs), tumor-associated macrophages (TAMs), and myeloid-derived suppressor cells (MDSCs) (13). CD4^+^ T cells differentiate into Th1 and Th2 subsets, with Th1 cells mediating antitumor immunity via IFN-γ and TNF-α, whereas Th2 cells promote oncogenesis through IL-4 and IL-5 (14). A Th2-skewed immune milieu, characterized by increased IL-4, IL-5, and IL-10, is frequently observed in affected patients (15). IL-10, in particular, exerts immunosuppressive effects primarily through activation of the JAK1/STAT3 pathway, which impairs dendritic cell and macrophage maturation, suppresses co-stimulatory molecule expression (CD80/CD86 and MHC-II), and diminishes proinflammatory cytokine secretion (16–18). These changes result in defective priming and expansion of cytotoxic CD8^+^ T lymphocytes, thereby fostering an immune-privileged tumor niche (19, 20). Concurrently, IL-10–driven STAT3 activation facilitates regulatory T cell differentiation, reinforcing immune tolerance and enabling tumor immune evasion (21, 22). Notably, neutralization of Th2-associated IL-10 has been shown to enhance the therapeutic efficacy of BCG immunotherapy (14, 23, 24). CTLs eliminate malignant cells through perforin–granzyme cytotoxicity and Fas–FasL signaling, with tumor-specific neoantigens augmenting their activity (25). In addition, CD8^+^ T cells induce ferroptosis via IFN-γ, thereby promoting antigen cross-presentation (26). Importantly, immune cell density and spatial organization within bladder tumors are heterogeneous (27, 28). Formation of tertiary lymphoid structures (TLS) at the tumor-stroma interface is associated with augmented antigen presentation, a favorable CD8^+^/Treg ratio, and improved patient survival, whereas an immune-excluded phenotype characterized by CD8^+^ T cells restricted to the tumor periphery without core infiltration is often linked to poor responses to immune checkpoint inhibitors (29, 30).

**Figure 1:**
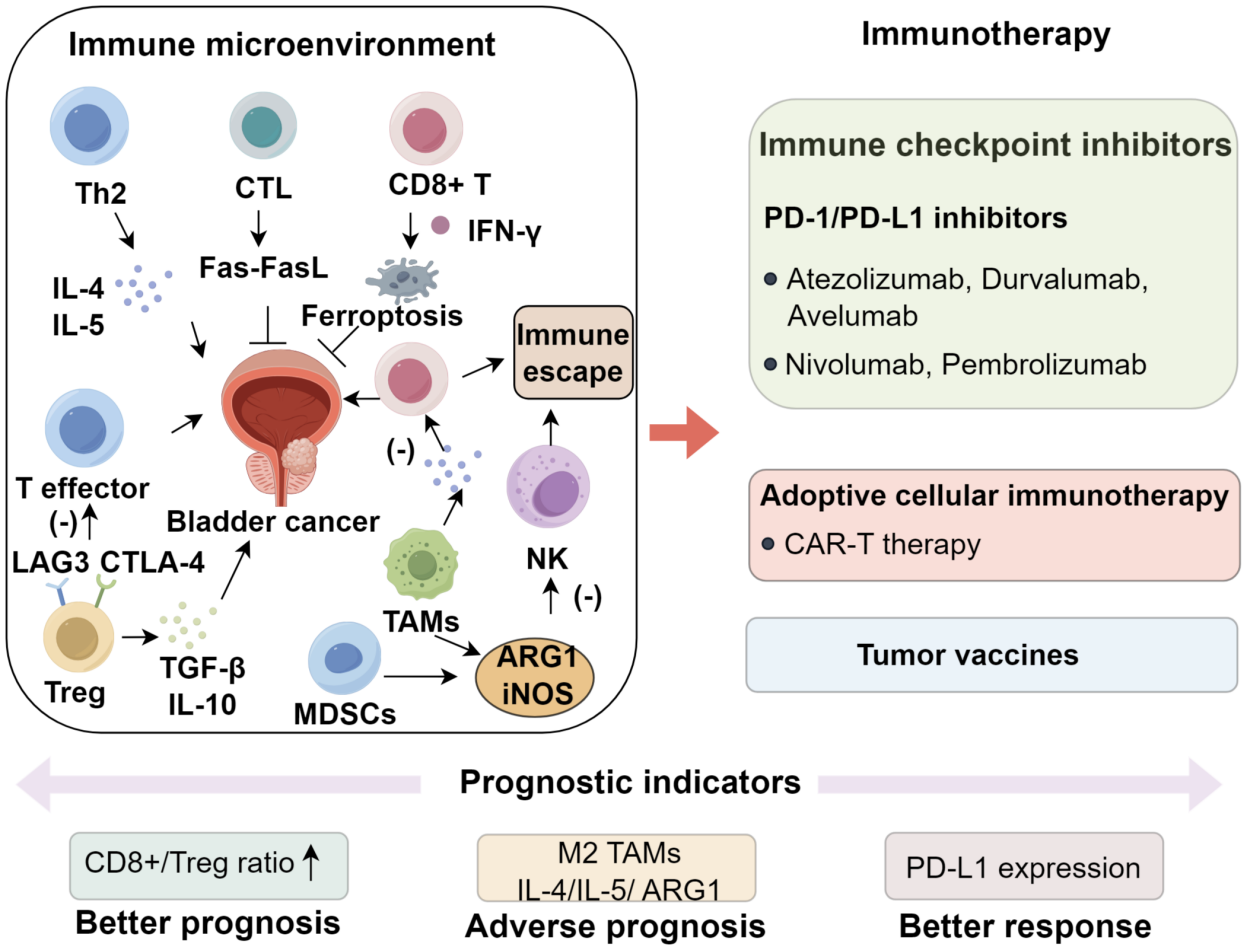
Immune Microenvironment and Immunotherapy of Bladder Cancer.

Regulatory T cells (Tregs) suppress effector T-cell activity through the secretion of immunosuppressive cytokines, including transforming growth factor−β (TGF−β) and IL−10, and by expressing inhibitory receptors such as CTLA−4 and LAG3, both of which are associated with BCG resistance and early disease recurrence (31, 32). Additional checkpoint receptors, notably TIM−3 and TIGIT, are frequently upregulated on Tregs and exhausted CD8^+^ T cells within the bladder TME, where they foster an immunosuppressive milieu and contribute to therapeutic resistance (33, 34). A high CD8^+^/Treg ratio has been linked to improved prognosis (35, 36). MDSCs further impair antitumor immunity by suppressing T- and natural killer (NK)-cell function through arginase−1 (ARG1) and inducible nitric oxide synthase (iNOS), while also exerting profound metabolic constraints on cytotoxic lymphocytes (37–39). ARG1 depletes extracellular L−arginine, diminishing CD3ζ chain expression and TCR signaling in T cells, whereas iNOS generates nitric oxide that forms peroxynitrite, leading to nitration of TCR components and subsequent T−cell apoptosis (40–42). These mechanisms collectively suppress CD8^+^ T−cell proliferation and cytotoxicity, creating an immunosuppressive niche that favors tumor progression and correlates strongly with advanced disease and poor clinical outcomes (23, 43, 44). TAMs, particularly the M2-polarized subset, are key orchestrators of this suppressive TME (45, 46). IL−4 and IL−13 secreted by Th2 cells activate STAT6 in macrophages, driving M2 polarization (47). M2−TAMs secrete VEGF, which promotes angiogenesis and tumor vascularization, and TGF−β, which facilitates extracellular matrix remodeling, invasion, and cytotoxic immune suppression (48). In addition, they produce IL−10 and ARG1, reinforcing immune tolerance by dampening effector T−cell function and promoting Treg expansion (23, 43). These mechanisms collectively contribute to tumor progression, immune evasion, and resistance to immunotherapy. Furthermore, PD-1/PD-L1 interactions between immune and tumor or stromal cells are central to local immune tolerance (49). Other checkpoint molecules including CTLA-4, LAG3 and TIGIT represent additional therapeutic targets currently under active investigation (50). Together, these immune components constitute a dynamic ecosystem where the balance between antitumor immunity mediated by factors such as CD8^+^ T cells and tertiary lymphoid structure formation, and immunosuppressive mechanisms involving regulatory T cells, myeloid-derived suppressor cells, tumor-associated macrophages and checkpoint engagement dictates disease evolution and therapeutic outcomes (51, 52). Elucidating these complex immune interactions provides a strong rationale for developing immune checkpoint blockade, adoptive cell therapy and combinatorial immunotherapeutic strategies in bladder cancer (**Figure 1**).

## 3 Immunological diagnosis of bladder cancer

Histopathological evaluation remains the gold standard for diagnosing urothelial carcinoma, with cystoscopy serving as the principal modality for both preoperative assessment and postoperative surveillance (53). Recent advances have introduced non-invasive immunodiagnostic strategies for urothelial carcinoma, notably assays for nuclear matrix protein−22 (NMP−22), bladder tumor antigen (BTA), and urinary cytology–based markers (uCyt^+^) (54–56). NMP-22 is a urinary biomarker overexpressed in affected patients, exhibits 52–59% sensitivity and 87–89% specificity (55, 56). The BTAstat assay achieves 64–69% sensitivity and 73–77% specificity, whereas the ELISA-based BTA−TRAK test shows 66% and 69%, with improved detection of high−grade tumors (57). uCyt^+^ identifies tumor-associated proteins in exfoliated urinary cells (73% sensitivity, 66% specificity), thereby reducing the need for unnecessary cystoscopy (58). Importantly, immunomagnetic enrichment coupled with immunofluorescence detection of circulating tumor cells (CTCs) demonstrates 35% sensitivity and 97% specificity for diagnosing urothelial malignancies, with CTC presence independently predicting unfavorable prognosis (59). Beyond simple enumeration, the phenotypic profiling of CTCs has revealed that PD-L1 expression on CTCs may serve as a dynamic biomarker of adaptive immune resistance (60). PD-L1–positive CTCs can directly suppress cytotoxic T cell activity, mirroring the tumor microenvironment’s immunosuppressive mechanisms.

## 4 Emerging immunotherapeutic strategies for bladder cancer

### 4.1 Intravesical BCG immunotherapy

Intravesical BCG administration remains the gold standard therapy for non-muscle invasive urothelial carcinoma. Its immunomodulatory effects are mediated by multiple mechanisms. Bacterial cell wall components, including antigen 85, bind to urothelial fibronectin and promote phagocytosis by antigen-presenting cells and malignant cells (61). Microbial recognition relies critically on pattern recognition receptors such as TLR2, TLR4, and TLR9 (62, 63). In addition to exerting direct cytotoxic effects, BCG induces the release of inflammatory mediators (IL-6, IL-8, TNF-α, GM-CSF), which recruit immune effector cells including T lymphocytes, B cells, and dendritic cells. Secondary cytokines such as IL-1β, IL-2, IFN-γ, and TRAIL subsequently activate innate and adaptive immune pathways, ultimately resulting in tumor cell apoptosis (64, 65). Current investigative efforts focus on three key domains: mechanistic elucidation, predictive biomarker discovery, and therapeutic optimization. Clinical parameters such as tumor burden, histological grade, and prior recurrence patterns influence therapeutic response (66). Moreover, molecular biomarkers (p53, retinoblastoma protein, survivin expression) and immunological parameters (urinary immune cell profiles) are emerging as promising predictive potential (66, 67). Notably, increased urinary regulatory T cell counts following BCG instillation associate with diminished therapeutic efficacy (68). The CyPRIT trial established a nine-cytokine signature (incorporating IL-2, IL-6, IFN-γ) with 85.5% predictive accuracy for recurrence (66). Innovative strategies to improve BCG efficacy include the development of genetically modified BCG strains (69) and combinatorial approaches with immunomodulators, particularly immune checkpoint inhibitors, which hold the potential to redefine the therapeutic standard for non-muscle-invasive disease (70).

### 4.2 The application of ICIs in bladder cancer management

#### 4.2.1 ICIs in advanced bladder cancer (platinum-refractory)

Therapeutic strategies for cisplatin-ineligible locally advanced or metastatic urothelial carcinoma now incorporate PD-L1 blockers (Atezolizumab, Durvalumab, Avelumab) and PD-1 antagonists (Nivolumab, Pembrolizumab) as secondary interventions (71–73). First-line approval has been granted to pembrolizumab and atezolizumab for PD-L1-positive cases or patients unsuitable for platinum-based regimens (74, 75). The advent of ICIs has revolutionized bladder cancer management, with PD-1/PD-L1 and CTLA-4 inhibitors representing the most clinically validated immunotherapies. Translational research concurrently focuses on identifying predictive biomarkers and managing immune-mediated adverse events (irAEs) (76). Preclinical evidence suggests selective targeting of CX-072 toward PD-L1-expressing malignancies, supported by early-phase data confirming its safety and efficacy in treatment-refractory solid tumors (77). Emerging agents targeting alternative immune checkpoints, including LAG3 and killer immunoglobulin-like receptors (KIR), are under investigation. LAG3 regulates T-cell function and exhibits antitumor activity, with compounds like BMS-986016 and LAG-525 showing promising early results (78).

For platinum-resistant metastatic urothelial carcinoma, ICIs constitute the therapeutic mainstay. KEYNOTE-045 demonstrated superior efficacy of pembrolizumab versus chemotherapy, achieving a 21.1% response rate and 10.3-month median survival. Enhanced outcomes (8.0-month survival) were noted in the PD-L1-high (≥10%) cohort, coupled with fewer severe toxicities (40). Long-term analysis confirmed enduring survival benefits (79). Similarly, IMvigor211 reported improved median OS (8.6 months) and lower severe toxicity rates with atezolizumab (10), with sustained survival advantages at 30 months (80–82). In CheckMate 275, nivolumab achieved an ORR of 19.6%, with differential responses across PD-L1 subgroups (28.4%, 23.8%, and 16.1%), alongside 8.6-month median survival and 40% 1-year survival, with 18% experiencing grade 3~4 toxicities (83, 84). Other PD-L1 inhibitors, including durvalumab and avelumab, exhibited comparable efficacy (85, 86). The PD-1 inhibitor tislelizumab yielded a 24% ORR, median OS of 9.8 months, and median progression-free survival (PFS) of 2.1 months, with one-year OS and PFS rates of 43% and 20% (87). CheckMate 032 evaluated nivolumab-ipilimumab combinations, revealing ORRs of 25.6% (nivolumab monotherapy), 26.9% (low-dose combination), and 38.0% (high-dose combination), with corresponding survival durations of 9.9, 7.4, and 15.3 months (88). Recent findings indicate a 37% response rate in rare urogenital malignancies with dual checkpoint blockade, though heightened irAEs necessitate careful patient selection (89) (**Supplementary Table S1**). These findings establish PD-1/PD-L1 inhibitors as standard second-line therapy for advanced platinum-refractory bladder cancer.

#### 4.2.2 ICIs for chemotherapy-naïve advanced bladder cancer

For cisplatin-ineligible patients with untreated advanced/metastatic bladder cancer, ICIs provide a non-chemotherapy option. KEYNOTE-052 assessed pembrolizumab in cisplatin-ineligible patients, reporting a 24% ORR and 67% six-month OS rate (90). Five-year data indicated median OS of 11.3 months, with PD-L1-high (CPS ≥10) patients exhibiting superior outcomes (OS: 18.5 months; ORR: 47.3%) (91). IMvigor210 documented a 23% ORR, median PFS of 2.7 months, and median OS of 15.9 months with atezolizumab (92, 93). KEYNOTE-361 detected no PFS improvement with pembrolizumab-chemotherapy versus chemotherapy alone (8.3 months), though pembrolizumab monotherapy correlated with higher durable response rates (52.0% at 18 months) (94, 95). IMvigor-130 demonstrated enhanced PFS (8.2 months) and OS (16.0 months) with atezolizumab-chemotherapy (94). Both trials highlighted reduced survival in low PD-L1 patients, prompting EMA and FDA to restrict ICIs to cisplatin-ineligible, high PD-L1 patients (96). Suboptimal outcomes in PD-L1-low subgroups prompted regulatory restrictions to cisplatin-ineligible, PD-L1-high populations (97), but DANUBE showed no significant efficacy difference between durvalumab ± tremelimumab and chemotherapy (98). Preclinical models support dual checkpoint inhibition (99), yet DANUBE revealed no OS benefit with durvalumab ± tremelimumab versus chemotherapy (100, 101). Maintenance immunotherapy seeks to prolong clinical responses while mitigating chemotherapy-induced toxicity. In the maintenance setting after initial chemotherapy, the phase III JAVELIN Bladder 100 trial established avelumab’s superiority, with median OS of 21.4 months. Avelumab exhibited a median PFS of 5.7 months in PD-L1-positive subgroup (102, 103). In contrast, pembrolizumab maintenance (phase II) improved PFS (5.4 months) and ORR (23%) without better OS benefit (22 months) (104).

#### 4.2.3 ICIs in muscle-invasive disease

In contrast to metastatic disease, MIBC is treated with a curative intent. Here, ICIs are evaluated as neoadjuvant, adjuvant, or part of bladder-preserving strategies. While cisplatin-based neoadjuvant chemotherapy remains standard for MIBC, ICIs offer a less toxic alternative. PURE-01 reported a 42% pathological complete response (pT0) rate with pembrolizumab, escalating to 54.3% in PD-L1-high patients (105). At 23-month follow-up, 24-month event-free survival was 71.7% (106). ABACUS (phase II) observed a 31% pT0 rate with atezolizumab (107, 108), while pembrolizumab plus gemcitabine-cisplatin achieved pT0N0 in 36% (109). Dual ICIs (nivolumab-ipilimumab) showed a 46% pT0 rate but frequent high-grade toxicity (110). Durvalumab plus Tremelimumab achieved 37.5% pT0 with 21% grade 3+ adverse events (111). Durvalumab-tremelimumab yielded 37.5% pT0 with manageable toxicity (82). Adjuvant nivolumab in CheckMate 274 doubled median disease-free survival (DFS: 20.8 vs. 10.8 months) without compromising health-related quality of life (HRQoL) (112, 113). Conversely, IMvigor010 reported no DFS/OS benefit with adjuvant atezolizumab, underscoring the need for further validation (114). Radical cystectomy remains the gold standard for MIBC, offering 5-year survival rates approaching 66%. However, the procedure carries substantial perioperative morbidity and adversely impacts patients’ quality of life (115, 116). Consequently, organ-sparing multimodal therapies have gained traction, particularly with the integration of ICIs. Radiotherapy has demonstrated immunomodulatory effects, including expansion of T-cell receptor repertoires, PD-L1 upregulation, and abscopal tumor regression (117, 118). The IMMUNOPRESERVE-SOGUG phase II trial investigated durvalumab and tremelimumab combined with radiotherapy following transurethral resection (TURBT) in MIBC patients. This chemotherapy-free regimen achieved 81% complete response (CR) rates, 73% 1-year bladder-intact disease-free survival (BIDFS), and 87% 1-year overall survival (OS), with grade ≥3 adverse events occurring in 31% of participants (119). Similarly, pembrolizumab with chemoradiation yielded 77% 1-year BIDFS and 80% CR at 12 weeks, albeit with 35% grade ≥3 toxicities (120). An alternative approach using nivolumab plus gemcitabine-cisplatin (GC) chemotherapy resulted in 48% CR, 92.4% 1-year OS, and 78% 1-year BIDFS among responders (121). These findings underscore the potential of immunotherapy-based bladder preservation strategies.

#### 4.2.4 Immunotherapy in non-muscle invasive bladder cancer

For high-risk NMIBC, the standard of care involves TURBT followed by intravesical Bacillus Calmette-Guérin (BCG) immunotherapy. Nevertheless, up to 50% of patients develop recurrence or BCG resistance within five years (122). While RC is an option for BCG-refractory disease, its associated risks necessitate alternative non-surgical interventions (123). Emerging evidence indicates that repeated BCG instillations, while initially stimulating anti-tumor immunity, can eventually induce adaptive immune resistance (124, 125). Chronic BCG exposure promotes sustained PD-L1 expression on tumor cells and infiltrating myeloid populations, thereby inhibiting cytotoxic T cell activity and creating an immunosuppressive microenvironment that underlies BCG treatment failure (126). This biological shift provides a strong rationale for targeting the PD-1/PD-L1 axis in BCG-unresponsive NMIBC. Emerging evidence implicates PD-1/PD-L1 axis activation in BCG resistance, with elevated PD-L1 expression observed in refractory tumors (127). The KEYNOTE-057 trial evaluated pembrolizumab in BCG-unresponsive NMIBC, demonstrating a 41% pathological CR at 3 months, with a median response duration of 16.2 months. Notably, no progression to muscle-invasive or metastatic disease occurred, and 3-year OS rates reached 91%. Grade III-IV toxicities were reported in 12.7% of patients (128). Based on these outcomes, ESMO guidelines endorse pembrolizumab for BCG-refractory NMIBC patients ineligible for or declining RC (113). Similarly, the SWOG S1605 trial reported a 41% CR at 3 months with atezolizumab, alongside a median response duration of 16.5 months. The 18-month event-free survival rate was 29%, with 12.3% grade III-IV adverse events (129, 130). Both agents exhibit comparable efficacy, with ongoing studies expected to refine their roles in clinical practice.

Recent advances in adoptive cell transfer have highlighted CAR-T cell therapy as a novel therapeutic strategy for treating solid malignancies such as bladder cancer (131). Preclinical investigations have provided evidence supporting the utility of CAR-T cells in BC models. In one study, Grunewald and colleagues reported that CAR-T cells directed against EGFR and CD44V6 effectively induced BC cell lysis, with decitabine, an inhibitor of DNA methyltransferase, further augmenting their antitumor activity (132). Another preclinical evaluation revealed that CAR-T cells targeting MUC1 exhibited cytotoxic effects on BC-derived organoids (133). Additionally, multiple clinical trials are currently evaluating CAR-T cell therapies in BC, focusing on antigens including PSMA, FRα, HER2, and ROR2 (134). Notably, SIA-CIgG, a glycosylated form of cancer-derived IgG, is abundantly expressed in BC and correlates with aggressive tumor behavior. Compared to HER2-targeting CAR-T cells, which have been widely tested in clinical settings, SIA-CIgG-specific CAR-T cells exhibit prolonged persistence and a more moderate tumor-lytic profile (135).

## 5 Conclusion

The immunotherapy revolution has fundamentally transformed bladder cancer management, offering new hope for patients across disease stages. Our review highlights several key advances: First, immune checkpoint inhibitors have established durable clinical benefits in advanced disease, with pembrolizumab demonstrating superior survival over chemotherapy in platinum-refractory patients and avelumab showing significant survival advantages as maintenance therapy. Second, bladder-preserving strategies combining ICIs with radiotherapy achieve impressive complete response rates (up to 81%) while maintaining organ function, challenging the traditional dominance of radical cystectomy for MIBC. Third, in NMIBC, PD-1 inhibitors provide effective salvage therapy for BCG-unresponsive disease, with pembrolizumab achieving 41% complete responses and 91% 3-year survival.

Critical challenges remain, including the need for better predictive biomarkers to guide patient selection, as PD-L1 expression and tumor mutational burden show imperfect correlation with treatment response. The management of immune-related adverse events requires ongoing refinement, particularly for combination therapies showing increased toxicity. Emerging approaches such as bispecific antibodies, CAR-T cell therapy, and novel ICIs targeting LAG-3 and KIR show preclinical promise but require further clinical validation. Future directions should focus on optimizing combination strategies, including ICI-chemotherapy-radiotherapy regimens, and developing next-generation biomarkers through multi-omics approaches. The integration of artificial intelligence for treatment response prediction and the development of personalized neoantigen vaccines represent exciting frontiers. As these innovations mature, they promise to further improve outcomes while reducing treatment morbidity, ultimately.

## References

[1] Zhou Y Zhang H Yan H Han P Liu Y. Immune landscape and prognostic significance of gene expression profiles in bladder cancer: insights from immune cell infiltration and risk modeling. Iran J Allergy Asthma Immunol. (2025) 24:519–32. doi: 10.18502/ijaai.v24i4.19132, PMID: 40696737

[2] Shen C Liu J Hu D Liu C Xie F Wang Y. Tumor-intrinsic ENO1 inhibition promotes antitumor immune response and facilitates the efficacy of anti-PD-L1 immunotherapy in bladder cancer. J Exp Clin Cancer Res. (2025) 44:207. doi: 10.1186/s13046-025-03464-x, PMID: 40665335 PMC12261641

[3] Saito R Taoka R Miki J Fukuokaya W Matsui Y Hatakeyama S. Efficacy of cisplatin-based neoadjuvant chemotherapy and risk factors for residual extravesical disease in muscle-invasive bladder cancer: insights from a nationwide cohort. Int J Clin Oncol. (2025). doi: 10.1007/s10147-025-02833-y, PMID: 40690100

[4] Mihai IM Wang G. Biomarkers for predicting bladder cancer therapy response. Oncol Res. (2025) 33:533–47. doi: 10.32604/or.2024.055155, PMID: 40109853 PMC11915070

[5] Compérat E Amin MB Cathomas R Choudhury A De Santis M Kamat A. Current best practice for bladder cancer: a narrative review of diagnostics and treatments. Lancet. (2022) 400:1712–21. doi: 10.1016/S0140-6736(22)01188-6, PMID: 36174585

[6] Willsmore ZN Coumbe BG Crescioli S Reci S Gupta A Harris RJ. Combined anti-PD-1 and anti-CTLA-4 checkpoint blockade: treatment of melanoma and immune mechanisms of action. Eur J Immunol. (2021) 51:544–56. doi: 10.1002/eji.202048747, PMID: 33450785

[7] Yang Z Chen Y Miao Y Yan H Chen K Xu Y. Elucidating stearoyl metabolism and NCOA4-mediated ferroptosis in gastric cancer liver metastasis through multi-omics single-cell integrative mendelian analysis: advancing personalized immunotherapy strategies. Discov Oncol. (2025) 16:46. doi: 10.1007/s12672-025-01769-z, PMID: 39812999 PMC11735723

[8] Shin MH Oh E Kim Y Nam DH Jeon SY Yu JH. Recent advances in CAR-based solid tumor immunotherapy. Cells. (2023) 12:1606. doi: 10.3390/cells12121606, PMID: 37371075 PMC10297050

[9] Wang L Zhou X Yan H Miao Y Wang B Gu Y. Deciphering the role of tryptophan metabolism-associated genes ECHS1 and ALDH2 in gastric cancer: implications for tumor immunity and personalized therapy. Front Immunol. (2024) 15:1460308. doi: 10.3389/fimmu.2024.1460308, PMID: 39328412 PMC11424447

[10] Kang HW Kim W-J Yun SJ. The role of the tumor microenvironment in bladder cancer development and progression. Translational Cancer Research (2017) 6. doi: 10.21037/tcr.2017.06.48

[11] Annels NE Simpson GR Pandha H. Modifying the non-muscle invasive bladder cancer immune microenvironment for optimal therapeutic response. Front Oncol. (2020) 10:175. doi: 10.3389/fonc.2020.00175, PMID: 32133299 PMC7040074

[12] Jiang L Jiang Y Zhou X Wang L Zhang S Jiang C. The key role of COA6 in pancreatic ductal adenocarcinoma: metabolic reprogramming and regulation of the immune microenvironment. J Cell Mol Med. (2025) 29:e70685. doi: 10.1111/jcmm.70685, PMID: 40596639 PMC12213452

[13] Joseph M Enting D. Immune responses in bladder cancer-role of immune cell populations, prognostic factors and therapeutic implications. Front Oncol. (2019) 9:1270. doi: 10.3389/fonc.2019.01270, PMID: 31824850 PMC6879653

[14] Pichler R Gruenbacher G Culig Z Brunner A Fuchs D Fritz J. Intratumoral Th2 predisposition combines with an increased Th1 functional phenotype in clinical response to intravesical BCG in bladder cancer. Cancer Immunol Immunother. (2017) 66:427–40. doi: 10.1007/s00262-016-1945-z, PMID: 28005163 PMC5359386

[15] Liu Y Fan M Xian S Hu P Zhang M Zhang X. RBP7 regulated by EBF1 affects th2 cells and the oocyte meiosis pathway in bone metastases of bladder urothelial carcinoma. Front Biosci (Landmark Ed). (2023) 28:189. doi: 10.31083/j.fbl2808189, PMID: 37664915

[16] Li J Lv Y Xue S Li W Zhang X. Ailanthone inhibits bladder cancer tumor and cell proliferation, epithelial-mesenchymal transition, and activation of the Janus kinase/signal transducer and activator of transcription 3 signaling pathway. Cytojournal. (2025) 22:16. doi: 10.25259/Cytojournal_166_2024, PMID: 40134568 PMC11932951

[17] Mazzoccoli L Cadoso SH Amarante GW de Souza MV Domingues R MaChado MA. Novel thalidomide analogues from diamines inhibit pro-inflammatory cytokine production and CD80 expression while enhancing IL-10. BioMed Pharmacother. (2012) 66:323–9. doi: 10.1016/j.biopha.2012.05.001, PMID: 22770990

[18] Xiong L Wang D Lin S Wang Y Luo M Gao L. Soluble CD83 inhibits acute rejection by up regulating TGF-β and IDO secretion in rat liver transplantation. Transpl Immunol. (2021) 64:101351. doi: 10.1016/j.trim.2020.101351, PMID: 33171217

[19] Lu TL Sher YP Chen HC Cheng WC Hsu LH Lee CC. Articulatin B chain induced dendritic cells maturation and driven type I T helper cells and cytotoxic T cells activation. Life Sci. (2022) 302:120635. doi: 10.1016/j.lfs.2022.120635, PMID: 35569571

[20] Fu C Tian G Duan J Liu K Zhang C Yan W. Therapeutic antitumor efficacy of cancer stem cell-derived DRibble vaccine on colorectal carcinoma. Int J Med Sci. (2021) 18:3249–60. doi: 10.7150/ijms.61510, PMID: 34400894 PMC8364449

[21] Wu D Wang Z. Gastric cancer cell-derived kynurenines hyperactive regulatory T cells to promote chemoresistance via the IL-10/STAT3/BCL2 signaling pathway. DNA Cell Biol. (2022) 41:447–55. doi: 10.1089/dna.2021.0936, PMID: 35353612 PMC9063152

[22] Xu Q Lin X Song L Ren Y Bai X Zhao X. et al: Trichinella spiralis excretory-secretory protein alleviates autoimmune thyroiditis by modulating Th17/Treg balance via the STAT3/STAT5 pathway. Acta Trop. (2025) 268:107706. doi: 10.1016/j.actatropica.2025.107706, PMID: 40562184

[23] Schneider AK Chevalier MF Derré L. The multifaceted immune regulation of bladder cancer. Nat Rev Urol. (2019) 16:613–30. doi: 10.1038/s41585-019-0226-y, PMID: 31501534

[24] Francesca B Meo M Giudice FD Scornajenghi CM Gazzaniga P Berardinis E. Exploring the utility of a NGS multigene panel to predict BCG response in patients with non-muscle invasive bladder cancer. Oncol Res. (2025) 33:723–31. doi: 10.32604/or.2024.056282, PMID: 40109859 PMC11915050

[25] Golstein P Griffiths GM. An early history of T cell-mediated cytotoxicity. Nat Rev Immunol. (2018) 18:527–35. doi: 10.1038/s41577-018-0009-3, PMID: 29662120

[26] Jiang X Stockwell BR Conrad M. Ferroptosis: mechanisms, biology and role in disease. Nat Rev Mol Cell Biol. (2021) 22:266–82. doi: 10.1038/s41580-020-00324-8, PMID: 33495651 PMC8142022

[27] Yoshihara K Ito K Kimura T Yamamoto Y Urabe F. Single-cell RNA sequencing and spatial transcriptome analysis in bladder cancer: Current status and future perspectives. Bladder Cancer. (2025) 11:23523735251322017. doi: 10.1177/23523735251322017, PMID: 40034247 PMC11864234

[28] Feng C Wang Y Song W Liu T Mo H Liu H. Spatially-resolved analyses of muscle invasive bladder cancer microenvironment unveil a distinct fibroblast cluster associated with prognosis. Front Immunol. (2024) 15:1522582. doi: 10.3389/fimmu.2024.1522582, PMID: 39759522 PMC11695344

[29] Zhao L Jin S Wang S Zhang Z Wang X Chen Z. Tertiary lymphoid structures in diseases: immune mechanisms and therapeutic advances. Signal Transduct Target Ther. (2024) 9:225. doi: 10.1038/s41392-024-01947-5, PMID: 39198425 PMC11358547

[30] Wu B Zhang B Li B Wu H Jiang M. Cold and hot tumors: from molecular mechanisms to targeted therapy. Signal Transduct Target Ther. (2024) 9:274. doi: 10.1038/s41392-024-01979-x, PMID: 39420203 PMC11491057

[31] Kim J-H Kim BS Lee S-K. Regulatory T cells in tumor microenvironment and approach for anticancer immunotherapy. Immune Netw. (2020) 20:e4. doi: 10.4110/in.2020.20.e4, PMID: 32158592 PMC7049587

[32] Koll FJ Banek S Kluth L Köllermann J Bankov K Chun FK. Tumor-associated macrophages and Tregs influence and represent immune cell infiltration of muscle-invasive bladder cancer and predict prognosis. J Transl Med. (2023) 21:124. doi: 10.1186/s12967-023-03949-3, PMID: 36793050 PMC9930232

[33] Liu Z Zhou Q Wang Z Zhang H Zeng H Huang Q. Intratumoral TIGIT(+) CD8(+) T-cell infiltration determines poor prognosis and immune evasion in patients with muscle-invasive bladder cancer. J Immunother Cancer. (2020) 8:e000978. doi: 10.1136/jitc-2020-000978, PMID: 32817209 PMC7430558

[34] Cai L Li Y Tan J Xu L Li Y. Targeting LAG-3, TIM-3, and TIGIT for cancer immunotherapy. J Hematol Oncol. (2023) 16:101. doi: 10.1186/s13045-023-01499-1, PMID: 37670328 PMC10478462

[35] Baras AS Drake C Liu J-J Gandhi N Kates M Hoque MO. The ratio of CD8 to Treg tumor-infiltrating lymphocytes is associated with response to cisplatin-based neoadjuvant chemotherapy in patients with muscle invasive urothelial carcinoma of the bladder. Oncoimmunology. (2016) 5:e1134412. doi: 10.1080/2162402X.2015.1134412, PMID: 27467953 PMC4910705

[36] Chi H Jiang L Zhou X Wang L Yang G Luo H. Editorial: Immune cell exhaustion: new challenges and opportunities in cancer therapy. Front Immunol. (2024) 15:1527428. doi: 10.3389/fimmu.2024.1527428, PMID: 39687607 PMC11646997

[37] Zhang H Ye YL Li MX Ye SB Huang WR Cai TT. CXCL2/MIF-CXCR2 signaling promotes the recruitment of myeloid-derived suppressor cells and is correlated with prognosis in bladder cancer. Oncogene. (2017) 36:2095–104. doi: 10.1038/onc.2016.367, PMID: 27721403

[38] Zhang Y Wang X Zhang R Wang X Fu H Yang W. MDSCs interactions with other immune cells and their role in maternal-fetal tolerance. Int Rev Immunol. (2022) 41:534–51. doi: 10.1080/08830185.2021.1938566, PMID: 34128752

[39] Jou E Chaudhury N Nasim F. Novel therapeutic strategies targeting myeloid-derived suppressor cell immunosuppressive mechanisms for cancer treatment. Explor Target Antitumor Ther. (2024) 5:187–207. doi: 10.37349/etat, PMID: 38464388 PMC10918238

[40] Fresno M Gironès N. Myeloid-derived suppressor cells in trypanosoma cruzi infection. Front Cell Infect Microbiol. (2021) 11:737364. doi: 10.3389/fcimb.2021.737364, PMID: 34513737 PMC8430253

[41] Navasardyan I Bonavida B. Regulation of T cells in cancer by nitric oxide. Cells. (2021) 10:2655. doi: 10.3390/cells10102655, PMID: 34685635 PMC8534057

[42] Tan C Li C Ge R Zhang W Wu Z Wang S. Mcl-1 downregulation enhances BCG treatment efficacy in bladder cancer by promoting macrophage polarization. Cancer Cell Int. (2025) 25:48. doi: 10.1186/s12935-025-03676-3, PMID: 39955585 PMC11830210

[43] Crispen PL Kusmartsev S. Immunotherapy: Mechanisms of immune evasion in bladder cancer. Cancer Immunol Immunother. (2020) 69:3–14. doi: 10.1007/s00262-019-02443-4, PMID: 31811337 PMC6949323

[44] Chevalier MF Trabanelli S Racle J Salomé B Cesson V Gharbi D. ILC2-modulated T cell-to-MDSC balance is associated with bladder cancer recurrence. J Clin Invest. (2017) 127:2916–29. doi: 10.1172/JCI89717, PMID: 28650339 PMC5531411

[45] Yu F Yu N Zhang L Xu X Zhao Y Cao Z. Emodin decreases tumor-associated macrophages accumulation and suppresses bladder cancer development by inhibiting CXCL1 secretion from cancer-associated fibroblasts. Nutr Cancer. (2025) 77:706–21. doi: 10.1080/01635581.2025.2480309, PMID: 40114381

[46] Deng X Huang Y Zhang J Chen Y Jiang F Zhang Z. Histone lactylation regulates PRKN-Mediated mitophagy to promote M2 Macrophage polarization in bladder cancer. Int Immunopharmacol. (2025) 148:114119. doi: 10.1016/j.intimp.2025.114119, PMID: 39854875

[47] Li X Hou R Ding H Gao X Wei Z Qi T. Mollugin ameliorates murine allergic airway inflammation by inhibiting Th2 response and M2 macrophage activation. Eur J Pharmacol. (2023) 946:175630. doi: 10.1016/j.ejphar.2023.175630, PMID: 36871665

[48] Zeng L He Y Huang F Jiang X Li YA Hu K. Nicotine and tar-multiple targets synergize to alter the immune micro-environment to induce prostate cancer. Discov Oncol. (2025) 16:1277. doi: 10.1007/s12672-025-03137-3, PMID: 40624438 PMC12234924

[49] Tang X Yu T Tong H Wu Y. Advancements in bladder cancer immunotherapy: a focus on intravesical approaches. Front Pharmacol. (2025) 16:1578146. doi: 10.3389/fphar.2025.1578146, PMID: 40697667 PMC12279809

[50] Li H Lu H Cui W Huang Y Jin X. A TP53-based immune prognostic model for muscle-invasive bladder cancer. Aging (Albany NY). (2020) 13:1929–46. doi: 10.18632/aging.202150, PMID: 33323544 PMC7880361

[51] Wang X Wang L. Research progress of ICIS in the treatment of bladder cancer. Panminerva Med. (2024). doi: 10.23736/S0031-0808.24.05102-4, PMID: 38381474

[52] Lv Z Hou J Wang Y Wang X Wang Y Wang K. Knowledge-map analysis of bladder cancer immunotherapy. Hum Vaccin Immunother. (2023) 19:2267301. doi: 10.1080/21645515.2023.2267301, PMID: 37903500 PMC10760393

[53] Ahmadi H Duddalwar V Daneshmand S. Diagnosis and staging of bladder cancer. Hematol Oncol Clin North Am. (2021) 35:531–41. doi: 10.1016/j.hoc.2021.02.004, PMID: 33958149

[54] Flores Monar GV Reynolds T Gordon M Moon D Moon C. Molecular Markers for Bladder Cancer Screening: An Insight into Bladder Cancer and FDA-Approved Biomarkers. Int J Mol Sci. (2023) 24:14374. doi: 10.3390/ijms241814374, PMID: 37762677 PMC10531979

[55] Kim J Kim WT Kim W-J. urology c: Advances in urinary biomarker discovery in urological research. Investig Clin Urol. (2020) 61:S8–S22. doi: 10.4111/icu.2020.61.S1.S8, PMID: 32055750 PMC7004831

[56] Batista R Vinagre N Meireles S Vinagre J Prazeres H Leão R. Biomarkers for bladder cancer diagnosis and surveillance: a comprehensive review. Diagnostics (Basel) (2020) 10:39. doi: 10.3390/diagnostics10010039, PMID: 31941070 PMC7169395

[57] Soorojebally Y Neuzillet Y Roumiguié M Lamy PJ Allory Y Descotes F. Urinary biomarkers for bladder cancer diagnosis and NMIBC follow-up: a systematic review. World J Urol. (2023) 41:345–59. doi: 10.1007/s00345-022-04253-3, PMID: 36592175

[58] Schmitz-Dräger C Bonberg N Pesch B Todenhöfer T Sahin S Behrens T. Replacing cystoscopy by urine markers in the follow-up of patients with low-risk non-muscle-invasive bladder cancer?-An International Bladder Cancer Network project. Urol Oncol. (2016) 34:452–9. doi: 10.1016/j.urolonc.2016.06.001, PMID: 27381893

[59] Zhang Z Fan W Deng Q Tang S Wang P Xu P. The prognostic and diagnostic value of circulating tumor cells in bladder cancer and upper tract urothelial carcinoma: a meta-analysis of 30 published studies. Oncotarget. (2017) 8:59527–38. doi: 10.18632/oncotarget.18521, PMID: 28938656 PMC5601752

[60] Tang M Zhang Z Wang P Zhao F Miao L Wang Y. Advancements in precision nanomedicine design targeting the anoikis-platelet interface of circulating tumor cells. Acta Pharm Sin B. (2024) 14:3457–75. doi: 10.1016/j.apsb.2024.04.034, PMID: 39220884 PMC11365446

[61] Zlotta AR Drowart A Van Vooren JP de Cock M Pirson M Palfliet K. Evolution and clinical significance of the T cell proliferative and cytokine response directed against the fibronectin binding antigen 85 complex of bacillus Calmette-Guerin during intravesical treatment of superficial bladder cancer. J Urol. (1997) 157:492–8. doi: 10.1016/S0022-5347(01)65185-1, PMID: 8996341

[62] Kaur A Kaushik D Piplani S Mehta SK Petrovsky N Salunke DB. TLR2 agonistic small molecules: detailed structure-activity relationship, applications, and future prospects. J Med Chem. (2021) 64:233–78. doi: 10.1021/acs.jmedchem.0c01627, PMID: 33346636

[63] Wu Y Du S Bimler LH Mauk KE Lortal L Kichik N. Toll-like receptor 4 and CD11b expressed on microglia coordinate eradication of Candida albicans cerebral mycosis. Cell Rep. (2023) 42:113240. doi: 10.1016/j.celrep.2023.113240, PMID: 37819761 PMC10753853

[64] Han J Gu X Li Y Wu Q. Mechanisms of BCG in the treatment of bladder cancer-current understanding and the prospect. BioMed Pharmacother. (2020) 129:110393. doi: 10.1016/j.biopha.2020.110393, PMID: 32559616

[65] Pettenati C Ingersoll MA. Mechanisms of BCG immunotherapy and its outlook for bladder cancer. Nat Rev Urol. (2018) 15:615–25. doi: 10.1038/s41585-018-0055-4, PMID: 29991725

[66] Kamat AM Li R O’Donnell MA Black PC Roupret M Catto JW. Predicting response to intravesical bacillus Calmette-Guérin immunotherapy: are we there yet? A systematic review. Eur Urol. (2018) 73:738–48. doi: 10.1016/j.eururo.2017.10.003, PMID: 29055653

[67] Cao R Yuan L Ma B Wang G Tian Y. Immune-related long non-coding RNA signature identified prognosis and immunotherapeutic efficiency in bladder cancer (BLCA). Cancer Cell Int. (2020) 20:1–18. doi: 10.1186/s12935-020-01362-0, PMID: 32607061 PMC7320553

[68] Chevalier MF Schneider AK Cesson V Dartiguenave F Lucca I Jichlinski P. Conventional and PD-L1-expressing regulatory T cells are enriched during BCG therapy and may limit its efficacy. Eur Urol. (2018) 74:540–4. doi: 10.1016/j.eururo.2018.06.045, PMID: 30033046

[69] Rodriguez D Goulart C Pagliarone AC Silva EP Cunegundes PS Nascimento IP. *In vitro* evidence of human immune responsiveness shows the improved potential of a recombinant BCG strain for bladder cancer treatment. Front Immunol. (2019) 10:1460. doi: 10.3389/fimmu.2019.01460, PMID: 31297119 PMC6607967

[70] Rentsch CA Derré L Dugas SG Wetterauer C Federer-Gsponer JR Thalmann GN. Building on a solid foundation: enhancing bacillus Calmette-Guerin therapy. Eur Urol Focus. (2018) 4:485–93. doi: 10.1016/j.euf.2018.10.010, PMID: 30415921

[71] Szabados B Kockx M Assaf ZJ van Dam PJ Rodriguez-Vida A Duran I. Final results of neoadjuvant atezolizumab in cisplatin-ineligible patients with muscle-invasive urothelial cancer of the bladder. Eur Urol. (2022) 82:212–22. doi: 10.1016/j.eururo.2022.04.013, PMID: 35577646

[72] Sekino Y Pham QT Kobatake K Kitano H Ikeda K Goto K. KIFC1 is associated with basal type, cisplatin resistance, PD-L1 expression and poor prognosis in bladder cancer. J Clin Med. (2021) 10:4837. doi: 10.3390/jcm10214837, PMID: 34768355 PMC8584707

[73] Einstein DJ Sonpavde G. Treatment approaches for cisplatin-ineligible patients with invasive bladder cancer. Curr Treat Options Oncol. (2019) 20:12. doi: 10.1007/s11864-019-0609-6, PMID: 30741358

[74] Nadal R Bellmunt J. Management of metastatic bladder cancer. Cancer Treat Rev. (2019) 76:10–21. doi: 10.1016/j.ctrv.2019.04.002, PMID: 31030123

[75] Resch I Shariat SF Gust KM. PD-1 and PD-L1 inhibitors after platinum-based chemotherapy or in first-line therapy in cisplatin-ineligible patients: Dramatic improvement of prognosis and overall survival after decades of hopelessness in patients with metastatic urothelial cancer. Memo. (2018) 11:43–6. doi: 10.1007/s12254-018-0396-y, PMID: 29606979 PMC5862914

[76] Autio KA Boni V Humphrey RW Naing AJCCR. Probody therapeutics: an emerging class of therapies designed to enhance on-target effects with reduced off-tumor toxicity for use in immuno-oncology. Clin Cancer Res. (2020) 26:984–9. doi: 10.1158/1078-0432.CCR-19-1457, PMID: 31601568 PMC8436251

[77] Naing A Thistlethwaite FC Spira AI Garcia-Corbacho J Randhawa M Eskens F. CX-072, a PD-L1 Probody therapeutic, as monotherapy in patients with advanced solid tumors: Preliminary results of PROCLAIM-CX-072. J Clin Oncol. (2019) 37:2513. doi: 10.1200/JCO.2019.37.15_suppl.2513

[78] Butt S-u Malik L. Pharmacology: Role of immunotherapy in bladder cancer: past, present and future. Cancer Chemother Pharmacol. (2018) 81:629–45. doi: 10.1007/s00280-018-3518-7, PMID: 29368051

[79] Bellmunt J de Wit R Vaughn DJ Fradet Y Lee JL Fong L. Pembrolizumab as second-line therapy for advanced urothelial carcinoma. N Engl J Med. (2017) 376:1015–26. doi: 10.1056/NEJMoa1613683, PMID: 28212060 PMC5635424

[80] Powles T Durán I van der Heijden MS Loriot Y Vogelzang NJ De Giorgi U. Atezolizumab versus chemotherapy in patients with platinum-treated locally advanced or metastatic urothelial carcinoma (IMvigor211): a multicentre, open-label, phase 3 randomised controlled trial. Lancet. (2018) 391:748–57. doi: 10.1016/S0140-6736(17)33297-X, PMID: 29268948

[81] Grande E Arranz J De Santis M Bamias A Kikuchi E Del Muro XG. Atezolizumab plus chemotherapy versus placebo plus chemotherapy in untreated locally advanced or metastatic urothelial carcinoma (IMvigor130): final overall survival analysis results from a randomised, controlled, phase 3 study. Lancet Oncol. (2024) 25:29–45. doi: 10.1016/S1470-2045(23)00540-5, PMID: 38101433

[82] Bajorin DF Witjes JA Gschwend JE Schenker M Valderrama BP Tomita Y. Adjuvant nivolumab versus placebo in muscle-invasive urothelial carcinoma. N Engl J Med. (2021) 384:2102–14. doi: 10.1056/NEJMoa2034442, PMID: 34077643 PMC8215888

[83] Sharma P Retz M Siefker-Radtke A Baron A Necchi A Bedke J. Nivolumab in metastatic urothelial carcinoma after platinum therapy (CheckMate 275): a multicentre, single-arm, phase 2 trial. Lancet Oncol. (2017) 18:312–22. doi: 10.1016/S1470-2045(17)30065-7, PMID: 28131785

[84] Siefker-Radtke AO Baron AD Necchi A Plimack ER Pal SK Bedke J. Nivolumab monotherapy in patients with advanced platinum-resistant urothelial carcinoma: Efficacy and safety update from CheckMate 275. Am Soc Clin Oncol. (2019) 26:5120–8. doi: 10.1200/JCO.2019.37.15_suppl.4524 PMC8166422 32532789

[85] Powles T O’Donnell PH Massard C Arkenau H-T Friedlander TW Hoimes CJ. Efficacy and safety of durvalumab in locally advanced or metastatic urothelial carcinoma: updated results from a phase 1/2 open-label study. JAMA Oncol. (2017) 3:e172411–e172411. doi: 10.1001/jamaoncol.2017.2411, PMID: 28817753 PMC5824288

[86] Patel MR Ellerton J Infante JR Agrawal M Gordon M Aljumaily R. Avelumab in metastatic urothelial carcinoma after platinum failure (JAVELIN Solid Tumor): pooled results from two expansion cohorts of an open-label, phase 1 trial. Lancet Oncol. (2018) 19:51–64. doi: 10.1016/S1470-2045(17)30900-2, PMID: 29217288 PMC7984727

[87] Ye D Liu J Zhou A Zou Q Li H Fu C. Tislelizumab in Asian patients with previously treated locally advanced or metastatic urothelial carcinoma. Cancer Sci. (2021) 112:305–13. doi: 10.1111/cas.14681, PMID: 33047430 PMC7780053

[88] Sharma P Siefker-Radtke A de Braud F Basso U Calvo E Bono P. Nivolumab alone and with ipilimumab in previously treated metastatic urothelial carcinoma: CheckMate 032 nivolumab 1 mg/kg plus ipilimumab 3 mg/kg expansion cohort results. J Clin Oncol. (2019) 37:1608–16. doi: 10.1200/JCO.19.00538, PMID: 31100038 PMC6879315

[89] McGregor BA Campbell MT Xie W Farah S Bilen MA Schmidt AL. Results of a multicenter, phase 2 study of nivolumab and ipilimumab for patients with advanced rare genitourinary Malignancies. Cancer. (2021) 127:840–9. doi: 10.1002/cncr.33328, PMID: 33216356 PMC13213840

[90] Balar AV Castellano D O’Donnell PH Grivas P Vuky J Powles T. First-line pembrolizumab in cisplatin-ineligible patients with locally advanced and unresectable or metastatic urothelial cancer (KEYNOTE-052): a multicentre, single-arm, phase 2 study. Lancet Oncol. (2017) 18:1483–92. doi: 10.1016/S1470-2045(17)30616-2, PMID: 28967485

[91] O’Donnell PH Milowsky MI Petrylak DP Hoimes CJ Flaig TW Mar N. Enfortumab vedotin with or without pembrolizumab in cisplatin-ineligible patients with previously untreated locally advanced or metastatic urothelial cancer. J Clin Oncol. (2023) 41:4107–17. doi: 10.1200/JCO.22.02887, PMID: 37369081 PMC10852367

[92] Balar AV Galsky MD Rosenberg JE Powles T Petrylak DP Bellmunt J. Atezolizumab as first-line treatment in cisplatin-ineligible patients with locally advanced and metastatic urothelial carcinoma: a single-arm, multicentre, phase 2 trial. Lancet. (2017) 389:67–76. doi: 10.1016/S0140-6736(16)32455-2, PMID: 27939400 PMC5568632

[93] Necchi A Joseph R Loriot Y Hoffman-Censits J Perez-Gracia J Petrylak D. Atezolizumab in platinum-treated locally advanced or metastatic urothelial carcinoma: post-progression outcomes from the phase II IMvigor210 study. Ann Oncol. (2017) 28:3044–50. doi: 10.1093/annonc/mdx518, PMID: 28950298 PMC5834063

[94] Powles T Csőszi T Özgüroğlu M Matsubara N Géczi L Cheng SY. Pembrolizumab alone or combined with chemotherapy versus chemotherapy as first-line therapy for advanced urothelial carcinoma (KEYNOTE-361): a randomised, open-label, phase 3 trial. Lancet Oncol. (2021) 22:931–45. doi: 10.1016/S1470-2045(21)00152-2, PMID: 34051178

[95] Loriot Y Alva AS Csőszi T Ozguroglu M Matsubara N Geczi L. *Post-hoc* analysis of long-term outcomes in patients with CR, PR, or SD to pembrolizumab (pembro) or platinum-based chemotherapy (chemo) as 1L therapy for advanced urothelial carcinoma (UC) in KEYNOTE-361. Am Soc Clin Oncol. (2021) 39:6. doi: 10.1200/JCO.2021.39.6_suppl.435

[96] Gourd E. EMA restricts use of anti-PD-1 drugs for bladder cancer. Lancet Oncol. (2018) 19:e341. doi: 10.1016/S1470-2045(18)30433-9, PMID: 29887223

[97] Koyama S Akbay EA Li YY Herter-Sprie GS Buczkowski KA Richards WG. Adaptive resistance to therapeutic PD-1 blockade is associated with upregulation of alternative immune checkpoints. Nat Commun. (2016) 7:10501. doi: 10.1038/ncomms10501, PMID: 26883990 PMC4757784

[98] Powles T van der Heijden MS Castellano D Galsky MD Loriot Y Petrylak DP. Durvalumab alone and durvalumab plus tremelimumab versus chemotherapy in previously untreated patients with unresectable, locally advanced or metastatic urothelial carcinoma (DANUBE): a randomised, open-label, multicentre, phase 3 trial. Lancet Oncol. (2020) 21:1574–88. doi: 10.1016/S1470-2045(20)30541-6, PMID: 32971005

[99] Herbst RS Arkenau HT Santana-Davila R Calvo E Paz-Ares L Cassier PA. Ramucirumab plus pembrolizumab in patients with previously treated advanced non-small-cell lung cancer, gastro-oesophageal cancer, or urothelial carcinomas (JVDF): a multicohort, non-randomised, open-label, phase 1a/b trial. Lancet Oncol. (2019) 20:1109–23. doi: 10.1016/S1470-2045(19)30458-9, PMID: 31301962

[100] Sternberg CN Petrylak DP Bellmunt J Nishiyama H Necchi A Gurney H. FORT-1: phase II/III study of rogaratinib versus chemotherapy in patients with locally advanced or metastatic urothelial carcinoma selected based on FGFR1/3 mRNA expression. J Clin Oncol. (2023) 41:629–39. doi: 10.1200/JCO.21.02303, PMID: 36240478 PMC9870218

[101] Knowles M Dyrskjøt L Heath EI Bellmunt J Siefker-Radtke AO. Metastatic urothelial carcinoma. Cancer Cell. (2021) 39:583–5. doi: 10.1016/j.ccell.2021.04.012, PMID: 33974855

[102] Powles T Park SH Voog E Caserta C Valderrama BP Gurney H. Avelumab maintenance therapy for advanced or metastatic urothelial carcinoma. N Engl J Med. (2020) 383:1218–30. doi: 10.1056/NEJMoa2002788, PMID: 32945632

[103] Vaddepally RK Kharel P Pandey R Garje R Chandra AB. Review of indications of FDA-approved immune checkpoint inhibitors per NCCN guidelines with the level of evidence. Cancers (Basel). (2020) 12:738. doi: 10.3390/cancers12030738, PMID: 32245016 PMC7140028

[104] Galsky MD Mortazavi A Milowsky MI George S Gupta S Fleming MT. Randomized double-blind phase II study of maintenance pembrolizumab versus placebo after first-line chemotherapy in patients with metastatic urothelial cancer. J Clin Oncol. (2020) 38:1797–806. doi: 10.1200/JCO.19.03091, PMID: 32271672 PMC7255983

[105] Necchi A Anichini A Raggi D Briganti A Massa S Lucianò R. Pembrolizumab as neoadjuvant therapy before radical cystectomy in patients with muscle-invasive urothelial bladder carcinoma (PURE-01): an open-label, single-arm, phase II study. J Clin Oncol. (2018) 36:3353–60. doi: 10.1200/JCO.18.01148, PMID: 30343614

[106] Bandini M Gibb EA Gallina A Raggi D Marandino L Bianchi M. Does the administration of preoperative pembrolizumab lead to sustained remission post-cystectomy? First survival outcomes from the PURE-01 study(☆). Ann Oncol. (2020) 31:1755–63. doi: 10.1016/j.annonc.2020.09.011, PMID: 32979511

[107] Powles T Kockx M Rodriguez-Vida A Duran I Crabb SJ van der Heijden MS. Clinical efficacy and biomarker analysis of neoadjuvant atezolizumab in operable urothelial carcinoma in the ABACUS trial. Nat Med. (2019) 25:1706–14. doi: 10.1038/s41591-019-0628-7, PMID: 31686036

[108] Szabados B Rodriguez-Vida A Durán I Crabb SJ van der Heijden MS Pous AF. Toxicity and surgical complication rates of neoadjuvant atezolizumab in patients with muscle-invasive bladder cancer undergoing radical cystectomy: updated safety results from the ABACUS trial. Eur Urol Oncol. (2021) 4:456–63. doi: 10.1016/j.euo.2020.11.010, PMID: 33612455

[109] Belay ED Abrams J Oster ME Giovanni J Pierce T Meng L. Trends in geographic and temporal distribution of US children with multisystem inflammatory syndrome during the COVID-19 pandemic. JAMA Pediatr. (2021) 175:837–45. doi: 10.1001/jamapediatrics.2021.0630, PMID: 33821923 PMC8025123

[110] van Dijk N Gil-Jimenez A Silina K Hendricksen K Smit LA de Feijter JM. Preoperative ipilimumab plus nivolumab in locoregionally advanced urothelial cancer: the NABUCCO trial. Nat Med. (2020) 26:1839–44. doi: 10.1038/s41591-020-1085-z, PMID: 33046870

[111] Gao J Navai N Alhalabi O Siefker-Radtke A Campbell MT Tidwell RS. Neoadjuvant PD-L1 plus CTLA-4 blockade in patients with cisplatin-ineligible operable high-risk urothelial carcinoma. Nat Med. (2020) 26:1845–51. doi: 10.1038/s41591-020-1086-y, PMID: 33046869 PMC9768836

[112] Witjes JA Galsky MD Gschwend JE Broughton E Braverman J Nasroulah F. Health-related quality of life with adjuvant nivolumab after radical resection for high-risk muscle-invasive urothelial carcinoma: results from the phase 3 CheckMate 274 trial. Eur Urol Oncol. (2022) 5:553–63. doi: 10.1016/j.euo.2022.02.003, PMID: 35288066 PMC10062393

[113] Powles T Bellmunt J Comperat E De Santis M Huddart R Loriot Y. Bladder cancer: ESMO Clinical Practice Guideline for diagnosis, treatment and follow-up☆. Ann Oncol. (2022) 33:244–58. doi: 10.1016/j.annonc.2021.11.012, PMID: 34861372

[114] Bellmunt J Hussain M Gschwend JE Albers P Oudard S Castellano D. Adjuvant atezolizumab versus observation in muscle-invasive urothelial carcinoma (IMvigor010): a multicentre, open-label, randomised, phase 3 trial. Lancet Oncol. (2021) 22:525–37. doi: 10.1016/S1470-2045(21)00004-8, PMID: 33721560 PMC8495594

[115] Stein JP Lieskovsky G Cote R Groshen S Feng AC Boyd S. Radical cystectomy in the treatment of invasive bladder cancer: long-term results in 1,054 patients. J Clin Oncol. (2023) 41:3772–81. doi: 10.1200/JCO.22.02762, PMID: 37499357

[116] Kretschmer A Grimm T Buchner A Jokisch F Ziegelmüller B Casuscelli J. Midterm health-related quality of life after radical cystectomy: A propensity score-matched analysis. Eur Urol Focus. (2020) 6:704–10. doi: 10.1016/j.euf.2019.02.017, PMID: 30853603

[117] Ngiow SF McArthur GA Smyth MJ. Radiotherapy complements immune checkpoint blockade. Cancer Cell. (2015) 27:437–8. doi: 10.1016/j.ccell.2015.03.015, PMID: 25873170

[118] Dovedi SJ Adlard AL Lipowska-Bhalla G McKenna C Jones S Cheadle EJ. Acquired resistance to fractionated radiotherapy can be overcome by concurrent PD-L1 blockade. Cancer Res. (2014) 74:5458–68. doi: 10.1158/0008-5472.CAN-14-1258, PMID: 25274032

[119] Garcia del Muro X Valderrama BP Medina A Cuellar MA Etxaniz O Gironés Sarrió R. Phase II trial of durvalumab plus tremelimumab with concurrent radiotherapy (RT) in patients (pts) with localized muscle invasive bladder cancer (MIBC) treated with a selective bladder preservation approach: IMMUNOPRESERVE-SOGUG trial. J Clin Oncol. (2021) 39:15. doi: 10.1200/JCO.2021.39.15_suppl.4505

[120] Balar AV Milowsky MI O’Donnell PH Alva AS Kollmeier M Rose TL. Pembrolizumab (pembro) in combination with gemcitabine (Gem) and concurrent hypofractionated radiation therapy (RT) as bladder sparing treatment for muscle-invasive urothelial cancer of the bladder (MIBC): A multicenter phase 2 trial. J Clin Oncol. (2021) 39:15. doi: 10.1200/JCO.2021.39.15_suppl.4504

[121] Galsky MD Daneshmand S Chan KG Dorff TB Cetnar JP O Neil B. Phase 2 trial of gemcitabine, cisplatin, plus nivolumab with selective bladder sparing in patients with muscle-invasive bladder cancer (MIBC): HCRN GU 16-257. J Clin Oncol. (2021) 39:15. doi: 10.1200/JCO.2021.39.15_suppl.4503

[122] Solsona E. Words of wisdom. Re: Final results of an EORTC-GU cancers group randomized study of maintenance bacillus Calmette-Guérin in intermediate- and high-risk Ta, T1 papillary carcinoma of the urinary bladder: one-third dose versus full dose and 1 year versus 3 years of maintenance. Eur Urol. (2014) 65:847–8. doi: 10.1016/j.eururo.2013.12.034, PMID: 24559905

[123] Saboya L Buosi K Silva T Candido E Morari J Velloso LA. Intradermal priming to intravesical Bacillus Calmette-Guérin in non-muscle invasive bladder cancer: A translational research and phase I clinical trial. Oncol Res. (2025) 33:1495–503. doi: 10.32604/or.2025.061812, PMID: 40486869 PMC12144654

[124] Lee A Floyd K Wu S Fang Z Tan TK Froggatt HM. BCG vaccination stimulates integrated organ immunity by feedback of the adaptive immune response to imprint prolonged innate antiviral resistance. Nat Immunol. (2024) 25:41–53. doi: 10.1038/s41590-023-01700-0, PMID: 38036767 PMC10932731

[125] Kates M Matoso A Choi W Baras AS Daniels MJ Lombardo K. Adaptive immune resistance to intravesical BCG in non-muscle invasive bladder cancer: implications for prospective BCG-unresponsive trials. Clin Cancer Res. (2020) 26:882–91. doi: 10.1158/1078-0432.CCR-19-1920, PMID: 31712383

[126] de Jong FC Kvikstad V Hoedemaeker RF van der Made ACJ van der Bosch TP van Casteren NJ. PD-L1 expression in high-risk non-muscle invasive bladder cancer is not a biomarker of response to BCG. World J Urol. (2025) 43:57. doi: 10.1007/s00345-024-05392-5, PMID: 39752014 PMC11698758

[127] Fukumoto K Kikuchi E Mikami S Hayakawa N Matsumoto K Niwa N. Clinical role of programmed cell death-1 expression in patients with non-muscle-invasive bladder cancer recurring after initial bacillus calmette-guérin therapy. Ann Surg Oncol. (2018) 25:2484–91. doi: 10.1245/s10434-018-6498-2, PMID: 29717423

[128] Balar AV Kamat AM Kulkarni GS Uchio EM Boormans JL Roumiguié M. Pembrolizumab monotherapy for the treatment of high-risk non-muscle-invasive bladder cancer unresponsive to BCG (KEYNOTE-057): an open-label, single-arm, multicentre, phase 2 study. Lancet Oncol. (2021) 22:919–30. doi: 10.1016/S1470-2045(21)00147-9 34051177

[129] Black PC Tangen C Singh P McConkey DJ Lucia S Lowrance WT. Phase II trial of atezolizumab in BCG-unresponsive non-muscle invasive bladder cancer: SWOG S1605 (NCT02844816). J Clin Oncol. (2021) 39:15. doi: 10.1200/JCO.2021.39.15_suppl.4541

[130] Black PC Tangen C Singh P McConkey DJ Lucia S Lowrance WT. Phase II trial of atezolizumab in BCG-unresponsive non-muscle invasive bladder cancer: SWOG S1605 (NCT02844816). Am Soc Clin Oncol. (2020) 84:536–44. doi: 10.1200/JCO.2020.38.15_suppl.5022 PMC10869634 37596191

[131] Zhang Z Li D Yun H Liu W Chai K Tong J. CAR-T cells in the treatment of urologic neoplasms: present and future. Front Oncol. (2022) 12:915171. doi: 10.3389/fonc.2022.915171, PMID: 35860578 PMC9292130

[132] Grunewald CM Haist C König C Petzsch P Bister A Nößner E. Epigenetic priming of bladder cancer cells with decitabine increases cytotoxicity of human EGFR and CD44v6 CAR engineered T-cells. Front Immunol. (2021) 12:782448. doi: 10.3389/fimmu.2021.782448, PMID: 34868059 PMC8637820

[133] Yu L Li Z Mei H Li W Chen D Liu L. Patient-derived organoids of bladder cancer recapitulate antigen expression profiles and serve as a personal evaluation model for CAR-T cells *in vitro* . Clin Transl Immunol. (2021) 10:e1248. doi: 10.1002/cti2.1248, PMID: 33552510 PMC7847802

[134] MacKay M Afshinnekoo E Rub J Hassan C Khunte M Baskaran N. The therapeutic landscape for cells engineered with chimeric antigen receptors. Nat Biotechnol. (2020) 38:233–44. doi: 10.1038/s41587-019-0329-2, PMID: 31907405

[135] Ding M Lin J Qin C Fu Y Du Y Qiu X. Novel CAR-T cells specifically targeting SIA-CIgG demonstrate effective antitumor efficacy in bladder cancer. Adv Sci (Weinh). (2024) 11:e2400156. doi: 10.1002/advs.202400156, PMID: 39178136 PMC11516049

